# Establishment of a Transformation Coupled *in vitro* End Joining Assay to Estimate Radiosensitivity in Tumor Cells

**DOI:** 10.3389/fonc.2020.01480

**Published:** 2020-08-20

**Authors:** Sarah Degenhardt, Kristin Dreffke, Urlike Schötz, Cordula Petersen, Rita Engenhart-Cabillic, Kai Rothkamm, Jochen Dahm-Daphi, Ekkehard Dikomey, Wael Yassin Mansour

**Affiliations:** ^1^Laboratory of Radiobiology and Experimental Radiooncology, University Medical Center Hamburg-Eppendorf, Hamburg, Germany; ^2^Department of Radiotherapy and Radiooncology, Philipps-University Marburg, Marburg, Germany; ^3^Department of Radiotherapy, University Medical Center Hamburg-Eppendorf, Hamburg, Germany; ^4^Mildred Scheel Cancer Career Center HaTriCS4, University Medical Center Hamburg-Eppendorf, Hamburg, Germany; ^5^Department of Tumor Biology, National Cancer Institute, Cairo University, Cairo, Egypt

**Keywords:** *in vitro* end joining assay, DSB repair pathway choice, radiosensitivity, HNSC, head and neck squamous cell carcinoma, classical NHEJ

## Abstract

Here, we present a modified *in vitro* end-joining (*EJ*) assay to quantify EJ capacity, accuracy as well as pathway switch to alternative end-joining (Alt-EJ) or single strand annealing (SSA). A novel transformation assay was established to specifically measure circular repair products, which correlate with classical EJ efficiency. The *EJ* assay was validated using EJ-deficient mammalian cell lines (Ku80, DNA-PKcs, LigIV, or XRCC4 mutants). A pathway switch to Alt-EJ and SSA was seen exclusively in Ku-deficient cells. Circular EJ product formation correlated with cell survival and DSB repair capacity after X-irradiation. Investigation of 14 HNSCC cell lines revealed differences in the total EJ capacity but a broader variation in the amount of circular repair products. Sequencing of repair junctions in HNSCC cells demonstrated a predominance of high-fidelity EJ and an avoidance of both Alt-EJ and SSA. A significant correlation was observed between the amount of circular repair products, repair of IR-induced DSB and radiosensitivity. Collectively, these data indicate that the presented *in vitro-EJ*-assay can not only estimate the repair capacity of cancer cells to enable the stratification into radiosensitive or radioresistant, but can also identify repair pathway deregulation such as a switch to Alt-EJ or SSA, which enables tumor targeting.

## Introduction

Ionizing radiation (IR) kills cells mainly by damaging DNA. Among IR-induced damages, DNA double-strand breaks (DSBs) are considered to be the most critical lesion (1). Although most of the induced DSBs will be efficiently repaired, few will either be un- or mis-repaired, leading to lethal chromosomal aberrations and eventually cell death (2). Therefore, a strong correlation between DSB repair capacity and cell survival after IR was reported (3–8). A minimal reduction in DSB repair capacity will profoundly impact the cellular radiosensitivity (9).

In humans, DSBs are repaired *via* two main pathways: non-homologous end-joining (NHEJ) and homologous recombination (HR). The central unit of NHEJ is the DNA-PK complex composed of the catalytic subunit (PKcs) and the heterodimer Ku70/80. Final ligation is performed by Artemis and Pol μ together with XRCC4, LigIV, and XLF (10). This repair is generally accurate or associated with deletion of only few base pairs. On the other hand, RAD51, BRCA1/2 are the central proteins for executing HR in an error-free mechanism (11). NHEJ involves the re-ligation of the two ends of a DSB without the use of significant homology, whereas HR uses homologous DNA sequences (i.e., sister chromatids as a template for repair). While NHEJ is active throughout all cell cycle phases, HR predominates in S-phase cells, when a sister chromatid is available.

The choice between these repair pathways is regulated by a functional hierarchy, which assures a fast and accurate repair of DSB (12, 13). According to this hierarchy, accurate NHEJ predominates and suppresses HR. However, it was also found that this hierarchy is often deregulated in tumor cells, with a switch to inaccurate pathways such as single strand annealing (SSA) or alternative end-joining (Alt-EJ). A shift to SSA was seen in the squamous cell carcinoma cell line SKX, where ATM-dependent DNA damage response was impaired (14, 15). Furthermore, a shift to Alt-EJ was reported quite often in bladder and head and neck tumor cells (16, 17). Previously, we observed such pathway switch in several tumor cell lines from different entities (18) and importantly also in tumor samples obtained from prostate cancer patients (19). So far, the factors causing a shift to the Alt-EJ are only partly understood. This shift occurs, when the initiation of the classical NHEJ (C-NHEJ) is hampered due to a defective Ku-DNA binding (13, 20). A complete shift to Alt-EJ was found for the Ku-deficient cell line xrs5 (12, 13, 20). A partial shift to Alt-EJ was found for prostate cancer cell lines over-expressing Bcl2, which will hinder Ku-DNA binding (21).

These defects in the central repair pathways NHEJ and HDR with a shift to other pathways are generally observed to result in a reduction of the overall DSB repair capacity and thereby causing an increase in cellular radiosensitivity (15, 21, 22). However, these defects in specific DSB repair pathways may also allow a highly specific targeting of tumors. It is shown for tumors being defective in HR that a specific radiosensitization can be achieved by combining radiation with an inhibition of PARP1 (23–25). On the other hand tumor cell lines being defective in Ku binding and therefore shifting to Alt-EJ can also be targeted by PARP1 inhibition plus IR (18, 21). Therefore, for an individualized tumor therapy it is of great relevance to identify not only the overall DSB repair capacity but also the specific DSB repair pathway used.

There are already several attempts to develop a robust assay to address DSB repair capacity. For example, it was shown that the expression of specific DSB repair proteins may be used as a surrogate of repair capacity. For example, Ku70 expression measured by immunohistochemistry (IHC) was found to have a strong association with tumor outcome after radiotherapy (26, 27). However, due to the scatter involved in IHC, such an analysis can only be determined for a huge number of tumor samples but not for an individual patient.

A great progress was made to determine the individual DSB repair capacity by measuring the amount of residual DSBs *via* γH2AX foci technique directly in freshly collected tumor biopsies using the *ex vivo* assay (19, 28, 29). An excellent association was obtained between the residual number of γH2AX foci as determined after an *ex vivo* exposure to 2 Gy and the known tumor response after radiotherapy (28).

DSB repair capacity can also be determined *via* an *in vitro* repair assay using cell free extracts (CFEs) and specific repair substrates (30–34). These repair substrates are generated by the digestion of plasmid DNA by specific restriction enzymes allowing to determine both the overall DSB repair capacity as well as the specific pathway used. So far, this analysis was mostly performed in cell lines with defined repair defects.

In the current study, we present a refined *in vitro* end-joining (*in vitro-EJ*) assay, which is capable of discriminating the end-joining efficiency of rodent and also mammalian cell lines with known DSB repair defects in a quantitative as well as a qualitative scale. This assay was used to analyze the end-joining efficiency in six mammalian cell lines with defined DSB repair defects but also in 14 different HNSCC cell lines. In addition, a transformation assay was developed from this *in vitro*-EJ assay to determine specifically the amount of circular repair products, which represent the accurate form of NHEJ. The amount of circular re-joined products was found to correlate with the overall DSB repair capacity as well as cell survival after IR. For the HNSCC cell lines, however, a broader scatter was seen, suggesting that DSB repair capacity may also be determined by other factors. Sequencing of repair junctions demonstrated a predominance of high-fidelity EJ mechanism in HNSCC cell lines.

## Materials and Methods

### Cell Culture and Irradiation

The experiments were performed with various rodent and mammalian cell lines defective in specific DSB repair genes: xrs5 (Ku80-deficient), XR-1 (XRCC4^−/−^), MO59J (DNA PKcs-deficient), 180BR (LIGIV^−/−^), and the respective wild-type counterparts CHO-K1, MO59K, and NFHH1. The 14 head and neck squamous cell carcinoma cell lines (HNSCC) used for this study are UM-SCC-3, UM-SCC-6, UM-SCC-9, UM-SCC-11b, and UM-SCC-47 (provided by T. E. Carey, Michigan, United States), UT-SCC-5, UT-SCC-8, UT-SCC-14 (provided by R. Grénman, Turku, Finland), UD-SCC-2 (provided by JP. Klussmann, Cologne, Germany), 93-VU-147T (provided by JP. De Winter, Amsterdam, The Netherlands), FaDu, SKX, Cal33, SAS, and XF354 (provided by M. Baumann, Dresden, Germany) cells. All cell lines were cultured in Dulbecco's modified Eagle's medium (DMEM) supplemented with 10% fetal bovine serum (FBS), 2 mM L-glutamine and 1% non-essential amino acids. Cells were incubated at 37°C in a humidified atmosphere containing 10% CO_2_.

Cells were irradiated by 0–6 Gy X-rays with 200 keV (15 mA) X-rays using RS225 research system (GLUMAY MEDICAL, Byfleet/UK) with additional filtration using a 0.5-mm Cu filter with a final dose rate of 0.8 Gy/min was used for irradiation.

### Preparation of Cell-Free Extracts

The preparation of cell-free extracts (CFEs) was performed as described previously (30, 33, 35). Cells grown to about 70% confluence were harvested by trypsinization (1 mg/ml Trypsin, 0.44 mg/ml EDTA) with a subsequent washing step in 1xPBS (without MgCl_2_ and CaCl_2_). Cell pellets were used either directly or were stored in FBS containing 10% Dimethylsulfoxid (DMSO) at liquid nitrogen. All following steps were performed at ice temperature. For membrane lysis cell pellets were washed twice in 1xPBS, centrifuged at 900 rpm for 5 min at 4°C and resuspended in four packed cell volumes (PCV) hypotonic lysis buffer (10 mM Tris-HCl, pH 8; 1 mM EDTA; 5 mM DTT) containing freshly added protease inhibitors (0.5 mM PMSF; 1.2 μM Leupetin; 1.2 μM Pepstatin A). Cells were incubated for up to 90 min till 80–90% of nuclei were free from cytoplasm, whereby fresh PMSF was added each 20 min.

When after 90 min no lysis of the cell membrane occurred the cell suspension was transferred to a Dounce homogenizer and cell membranes were mechanically destroyed by 20 strokes using a loos fitting pistil. Nuclear lysis and the release of DNA-bound proteins were performed by adding 4 PCV high salt buffer (50% Glycerol; 25% Sucrose; 50 mM Tris-HCl, pH 8; 10 mM MgCl_2_; 2 mM DTT) and 80% PCV 3.9 M ammonium sulfate (pH 7) and a subsequent incubation for at least 30 min at 4°C. Cell lysates were further centrifuged at 4°C, 3,070 g for 2 h to eliminate genomic DNA and insoluble components. Afterwards the volume of the supernatant was measured and proteins within were precipitated for 30 min using 0.33 g/ml Ammonium sulfate and 10 μL/g Ammonium sulfate 1N NaOH. Precipitates were pelletized by centrifugation for 30 min at 27,000 g and 2°C. Pellets were solubilized in dialysis buffer (30 mM Tris-HCl, pH 8; 90 mM KCl; 10 mM β-Na-Glycerophosphate, pH 7; 2 mM EGTA, pH 8.5; 1 mM EDTA, pH 8; 2 mM MgCl_2_; 20% Glycerol; 2 mM DTT; 0.5 mM PMSF; 1 μM Leupeptin; 1 μM Pepstatin A). For solubilization one 20th part of the volume of the protein solution before precipitation was used. The protein solution was dialyzed in two steps, the first overnight and the second for further 60 min. Finally CFEs were cleared by centrifugation for 5 min at 16,000 g and 4°C, aliquoted and snap-frozen in liquid nitrogen. One aliquot was used for protein measurement using the Pierce BCA Protein Assay Kit (Thermo Scientific, Bonn/Germany) following the manufacturer instructions.

### Preparation of NHEJ Substrates

Substrates for the *in vitro* NHEJ reaction were generated by digesting the pEJ-1200 or pEJSSA-1299 plasmid (Figure S1A) using the restriction endonucleases HindIII and PstI (New England Biolabs, Frankfurt a.M./Germany). After electrophoresis on 1% Agarose gels, the linearized form was isolated using the Wizard SV Gel and PCR Clean-Up Sytem (Promega, Mannheim/Germany) following the users instructions. DNA concentration and purity was measured using a NanoDrop 2000 (Thermo Scientific, Waltham, MA/USA).

### *In vitro* NHEJ Reaction

For *in vitro* NHEJ reactions 65 μg CFE was dialyzed for 30 min at 4°C against MOPSO dialysis buffer (50 mM MOPSO; 40 mM KCl; 10 mM MgCl_2_; 0.9 mM β-Mercaptoethanol) using microdialysis membranes (MF-Membrane Filters 0.025 μm VSWP, Merck Millipore, Billerica, MA/USA). Reactions were performed in a total volume of 30 μl together with 50 ng NHEJ substrate and 1x LNB buffer (0.1 M Tris pH 8.0; 12 mM MgCl_2_; 0.1 M KCl; 0.18 mM β-Mercaptoethanol; 1 mM ATP; 2 mM dNTPs; 0.5 mg/ml BSA) at 25°C for 4 h in an Arktik Thermal Cycler (Thermo Scientific, Waltham, MA/USA). Reactions were stopped by adding working TE stop solution (1% SDS; 20 mM EDTA; 40 mM Tris, pH 7.5) in a 1:1 ratio and a subsequent incubation at 65°C for 10 min. Reactions were stored at −20°C till analysis.

### Southern Blotting

The quantitative and qualitative analysis of *in vitro* NHEJ products was performed by Agarose gel electrophoresis followed by Southern blot. First proteins in the *in vitro* NHEJ reactions were digested with 2 mg/ml Proteinase K at 37°C for 30 min before heat inactivation at 65°C for 15 min. The digestion was performed in Loading buffer (1.41 mM Tris-HCl, pH 8; 28,2 mM EDTA, pH 8; 26.5% Glycerole; 0.0056% Bromphenolblue; 0.0056% XanthoCyan) which was added to the NHEJ reaction in a 1:1 ratio. Afterwards, samples were loaded on a 0.8% Agarose gel (1x TAE and 1 μg/ml Ethidium bromide). The gel was rinsed in 0.2 N HCl for 15–30 min to partially depurinate DNA. The complete denaturation of DNA was subsequently done by incubation of the gel for 30 min in denaturation buffer (0.6 M NaCl; 0.4 M NaOH). The DNA was transferred to a nylon membrane (Amersham Hybond-N, GE Healthcare, Freiburg/Germany) via vacuum blotting for 30 min. Subsequently the membrane-bound DNA was neutralized for 15 min in neutralization buffer (1 M NaCl; 0.5 M Tris-HCl, pH 7.2) and cross-linked for 1 h at 37°C.

For the detection of membrane-bound DNA, Amersham Gene Images AlkPhos Direct Labeling and Detection System (GE Healthcare, Freiburg/Germany) was used following the manufacturer instructions. The chemiluminescence signal was detected using either x-ray films (Amersham Hyperfilm ECL, GE Healthcare, Freiburg/Germany) or the ChemoCam Imager 3.2 (Intas Imaging Instruments, Göttingen/Germany). Quantification of all different NHEJ products was performed using either the Un-Scan-It Gel 6.1 software (Silk Scientific, Orem, UT/USA) or the Quantity One software (BIO-RAD, München/Germany). End joining efficiency was measured using the following formula,

$$\begin{array}{l} \text{End joining efficiency }\left(\text{EJ}\%\right)\\ =\frac{\text{Intensity of repair product}}{\text{Intensities of }\left(\text{all repair product }+\text{ linear form}\right)}. \end{array}$$

### Transformation Assay

A quantitative analysis of the circular *in vitro* NHEJ products is performed by transforming *E. coli* cells with the NHEJ reaction. Briefly, 6 μl NHEJ reaction (containing 5 ng DNA) were dialyzed for 30 min against ddH_2_O using micro-dialysis membranes (MF-Membrane Filters 0.025 μm VSWP, Merck Millipore, Billerica, MA/USA). The dialyzed DNA was transformed in electro-competent *E. coli* cells (TransforMax EC100, Epicenter, Madison, WI/USA) using *E. coli* Pulser (BIO-RAD, München/Germany) at 1.8 kV. After incubation for 1 h at 225 rpm and 37°C in LB medium, bacteria were plated on LB agar plates containing 50 μg/ml kanamycin before being incubated over night at 37°C. Then the bacterial colonies were counted and the number of colonies per 1 ng transformed DNA was calculated.

### Sequencing of Repair Junctions

A quantitative analysis of the circular *in vitro* NHEJ products was performed using transformation assay. Briefly, DNA was isolated from the end-joining reactions using “DNeasy Blood & Tissue Extraction” kit and was PCR-amplified using P1 (5′-GGC AAA TGG GCG GTA GGC GTG-3) and P2 (5′-GTC GGG CAT GGC GGA CTT GAA-3′) primers. The PCR reaction started with an initial denaturation at 96°C for 2 min, followed by an amplification step over 35 cycles denaturation at 96°C for 20 s, annealing at 68°C for 20 s and elongation at 72°C for 80 s, and a post-amplification elongation step at 72°C for 7 min. PCR products were purified and sub-cloned using the “Topo-TA-Cloning” kit, according to the manufacturer's instructions. The resulting vectors were transformed in electro-competent bacteria. Single bacterial colonies were picked and suspended in 25 μl of PCR reaction buffer (12.5 μl 2x PeqGold Mester-MixY, 1 μl primer P1, 1 μl primer P2, 10.5 μl ddH_2_O) and amplified as described above. For sequencing of the repair junctions, PCR products were purified and sent for sequencing to GATC Biotech (Konstanz/Germany).

### Colony Formation

Cellular survival was determined via colony formation assay as previously described (14). Briefly: Cells were cultured to 80% confluence, irradiated and plated as triplicates in one third conditioned medium in T25 cell culture flasks 14 h after irradiation. The number of plated cells ranged from 500 to 3,000 cells, depending on the plating efficiency (PE) of each individual cell line. After incubation for 10–16 days without medium change colonies were fixed in 70% ethanol and stained in 0.1% Crystal violet. Cellular survival was defined as the ability to form colonies containing at least 50 cells and surviving fractions (SF) were calculated by normalization to the plating efficiency of the un-irradiated control.

### Graphs and Statistics

Unless stated otherwise, experiments were independently repeated at least three times. Data points represent the mean ± SEM of all individual experiments. Statistical analysis, data fitting and graphics were performed with the GraphPad Prism 6.0 program (GraphPad Software).

## Results

### A Modified *in vitro-EJ* Assay Measuring the End-Joining Capacity in Mammalian Cells

**Figure 1:**
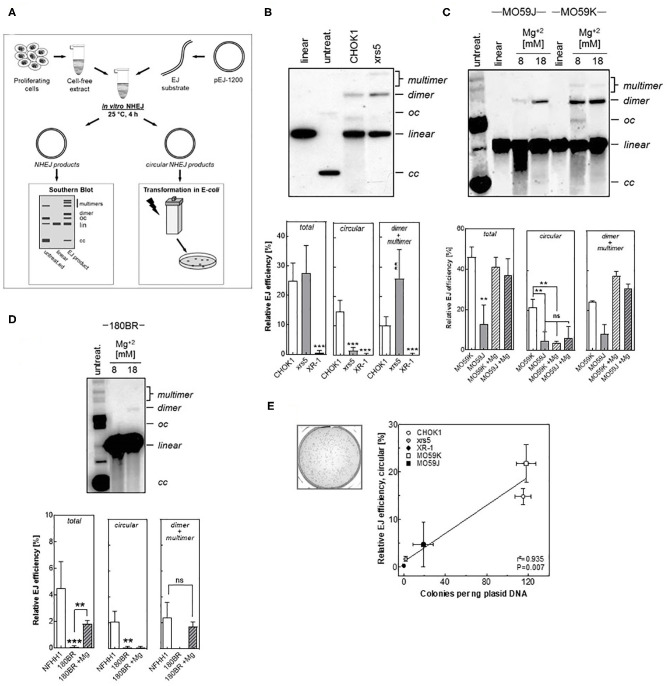
Detection of end joining efficiency using *in vitro-EJ* assay. **(A)** Experimental scheme showing the steps of the *in vitro-EJ* assay. **(B)** Upper panel: Representative Southern blot showing the different *in vitro* repair products in CHOK1 and xrs5 cells. Lower panel: Quantification of the indicated repair products shown in the upper panel. **(C,D)** Upper panels: Southern blotting of the *in vitro* repair products generated using cell extracts from **(C)** MO59K or MO59J and **(D)** 180BR cells in the presence of the indicated Mg2^+^ concentrations. Lower panels: Quantification of the experiment performed in the upper panels. Significance is indicated as **P < 0.001 and ***P < 0.0001. ns: not significant. **(E)** Left panel: Representative bacterial colonies generated upon transfecting *E.coli* with the repair products of CHO-K1 cells. Right panel: Correlation between number of colonies generated per 1 ng plasmid and the circular products detected (oc and cc) by the Southern blot. Pearson correlation coefficient (*r*^2^) and *p*-value are shown. Shown are mean ± SEM of at least three independent experiments. oc, open circle; cc, closed circle.

The currently described assay (Figure 1A) is based on the incubation of a linearized plasmid with cell free extract, which contains all soluble nuclear and cytoplasmic components in a physiologic and active condition. The linearized plasmid was generated by digestion of pEJ-1200 plasmid using HindIII and PstI restriction enzymes, generating non-cohesive DNA-ends (Figure S1A), which will only be repaired by an active repair process. The end joining reaction is performed at 25°C for 4 h in the presence of ATP and dNTPs. Different re-joining products could be generated through the joining of (i) HindIII- to PstI- ends (i.e., head-to-tail), (ii) HindIII- to HindIII- ends (i.e., head-to-head), or (iii) PstI- to PstI- ends (i.e., tail-to-tail) (Figure S1B). Under these conditions almost exclusively head to tail (H:T) products were generated (Figure S1C). All repair events including dimers, multimers and circular products were visualized by Southern blot (Figure S1D). Circular end-joining products were more generated at 25°C than at 37°C (Figure S1E), confirming previously reported data (35). This is attributed to the fact that at 37°C the nucleolytic activity within the CFE exceeds the end joining activity and thus, degradation of plasmids is faster than their repair (34). Additional optimizing efforts revealed that the optimal end-joining efficiency with a reasonable ratio between DNA repair and degradation was obtained with a DNA-protein ratio of 1:1000–1:800 (Figure S1F). Further experiments were performed using a ratio of 1:1000 (i.e., 65 μg CFE + 50 ng substrate).

### The *in vitro* Assay Detects the Different Efficiency of End-Joining in Mammalian Cell Lines Defective in DSB Repair

We investigated whether our *in vitro-EJ* assay is capable of discriminating NHEJ-deficiency at different levels. To that end, several mammalian cell lines with defined NHEJ-deficiency were employed including: (i) hamster cell lines with defects in either Ku80 (xrs5) or XRCC4 (XR-1) as well as their respective control cell line CHO-K1; (ii) the human glioblastoma DNA-PK-deficient MO59J cells and their wildtype cells MO59K and (iii) the human fibroblast line 180BR deficient in LIGIV together with a normal human fibroblast strain NFHH1. The pattern of the repair products was expectedly found to depend on the respective NHEJ-defect the cell line has. For the Ku80-defective xrs5 cells, although the quantity of the total end-joining repair products is similar to that of the control cell line, the amount of dimers and multimers was 1.6 times higher in xrs5 cells and the fraction of circular products was 9-fold lower compared to CHO-K1 cells (Figure 1B). This pattern was completely different in cell lines defective in NHEJ proteins down-stream of Ku. For example, DNA-PKcs-defective MO59J cells showed a 4.5- and 3- fold decrease in circular and dimers + multimers, respectively (Figure 1C). Similarly, a very strong reduction in the total end-joining activity with almost no circular or dimers + multimers were observed in both cells defective in the NHEJ ligation step (180BR and XR-1 cells) when compared to the respective controls (Figure 1D). These data confirm our previously published data that the repair is switched to Alt-EJ in xrs5 cells, which largely compensates the total repair deficiency in these cells, and such compensatory effect was not observed in cell lines with NHEJ-deficiencies downstream Ku (12, 13, 20).

Previously we reported that DSB repair is primarily carried out mainly by C-NHEJ in CHOK1, while in NHEJ-deficient cells the end-joining is performed by Alt-EJ (12, 13). Given that the circular products were not obvious in the NHEJ-defective cells, we speculated that these products are generated primarily by C-NHEJ. To test this assumption, we increased the Mg^+2^ concentrations to stimulate the activity of DNA-Ligase III, which is involved in Alt-EJ (36), and measured the *in vitro*-EJ in the NHEJ-defective MO59J cells. Supportive to our assumption, increasing Mg^+2^ concentration caused a strong reduction in the amount of circular products generated in NHEJ-proficient MO59K cells but had no effect on MO59J cells (Figure 1C). Of note, dimers and multimers were increased upon increasing Mg^+2^ concentrations in both strains. Similar data were obtained for the LIGIV-deficient cell line 180BR (Figure 1D). Together, these data indicate that the circular products result solely from C-NHEJ, while the dimers + multimers might arise from both C-NHEJ, Alt-EJ, and SSA.

### The Circular Repair Products Can Selectively Be Detected Using a Transformation Assay

Here, we sought to establish a fast- and cost- efficient transformation assay to determine the efficiency to specifically form circular repair products. To that end, all repair products were transformed into *E. coli* cells using electroporation. While all linear repair products are quickly degraded, only circular products will be propagated in bacterial cells enabling survival on kanamycin selection medium. Consequently, the number of bacterial colonies grown in this selection medium reflects the amount of circular products formed (Figure 1E). Analysis of the correlation between the relative end joining efficiency of circular products measured by Southern blot and the number of generated colonies from *in vitro* end joining reactions mediated by CHO-K1, xrs5, XR-1, MO59K, and MO59J cells revealed a highly significant correlation (*P* = 0.007) (Figure 1E). This confirms that the transformation assay can in fact be used to selectively detect the circular products.

### Analysis of Repair Fidelity Using *in vitro-EJ* Assay

**Figure 2:**
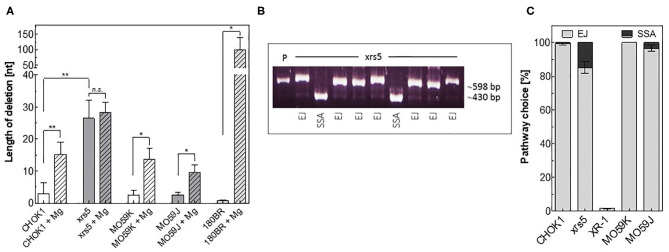
Detection of repair fidelity and switch using *in vitro-EJ* assay. **(A)** *In vitro* end joining repair junctions were sequenced to determine the deletion length in the indicated cell lines. **(B)** Examples of PCR amplification of repair junctions mediated by xrs5 extracts showing end joining (EJ) and SSA products. P: linearized plasmid. An exact 415-bp fragment indicates a SSA event. **(C)** Relative fractions of NHEJ events (gray) and SSA (black) in the indicated cell lines. Shown are mean ± SEM of at least three independent experiments. Significance is indicated as *P < 0.01 and **P < 0.001. ns, not significant.

We tested then whether our *in vitro* system is able to detect repair fidelity. Therefore, repair junctions mediated *in vitro* in CFEs from NHEJ-deficient and proficient cells were amplified and a total of 244 events were sequenced. Briefly, all *in vitro-EJ* repair products obtained from NHEJ-deficient MO59J, 180BR, and xrs5 cells or from their wildtype counterparts CHO-K1 and MO59K control cells were amplified by PCR. Thereafter, amplicons were sub-cloned via Topo-TA cloning, transformed into *E. coli* and the repair junctions of single clones was amplified, sequenced, and compared to the original sequence at the break site. In line with the data presented in Figure 1, we found that in xrs5 cells with a pathway switch to Alt-EJ, the repair junctions were associated with a significantly increased (*P* = 0.003) deletion length compared to their wild type CHOK1 cells (Figure 2A). Notably, the end-joining in other NHEJ-deficient cells (MO59J&180BR) is associated with short deletion lengths (Figure 2A), probably due to the presence of Ku proteins, which prevent the switch to Alt-EJ in these cells (13). Consistently, the deletion length is increased significantly in these cells upon increasing the Mg^+2^ concentration, which enables pathway switch to the inaccurate Alt-EJ (Figure 2A).

We next asked whether we can detect the repair shift to SSA using our *in vitro-EJ* system. To test this, the *in vitro-EJ* assay used here was performed with the end-joining substrate pEJSSA-1200 (12, 15), which enables the use of SSA in addition to NHEJ (Figure S1A, Figure 2B). Our data revealed that SSA was almost completely avoidable in wild type CHO-K1 and MO59K cell lines, confirming our previously published data (15). In contrast, SSA events were increased in xrs5 and MO59J cells (15 and 3%, respectively). For the XRCC4-deficient XR-1 cell line being strongly deficient in C-NHEJ no event performed by SSA was detected (Figures 2B,C). Collectively, these data reveal that the described *in vitro-EJ* assay can detect (i) total end joining efficiency, (ii) C-NHEJ efficiency, (iii) EJ repair fidelity and pathway switch to either Alt-EJ or SSA.

### The Circular Repair Products Reflect the Capacity of Repairing IR-Induced DSBs

**Figure 3:**
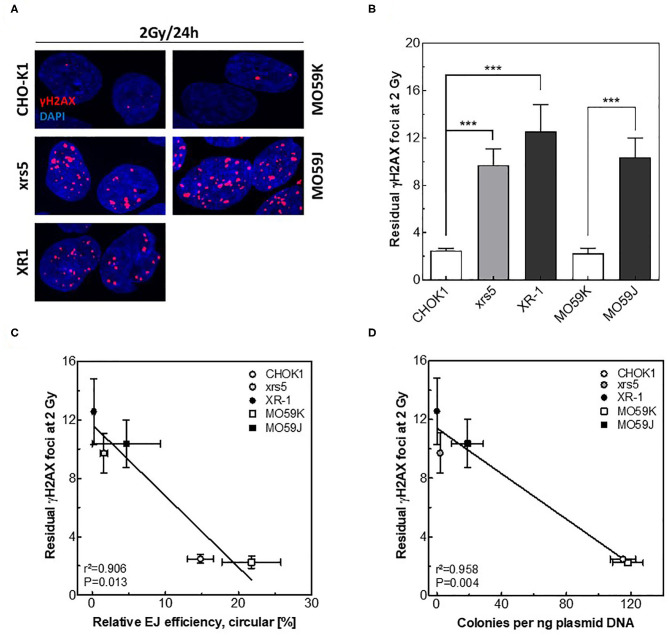
Circular repair products correlate with number of residual γH2AX foci after IR. **(A)** Representative IF images of γH2AX (red) foci at 24 h post 2 Gy in the indicated cell lines. DAPI was used to counterstain nuclei. **(B)** Quantification of experiment performed in A. Significance is indicated as ***P < 0.0001. **(C,D)** Correlation between the number of residual γH2AX foci post 2 Gy and relative efficiency of generating circular repair products measured by **(C)** Southern blot or **(D)** transformation assay in the indicated cell lines. Shown are mean ± SEM of at least three independent experiments.

Next, we tested whether the measured *in vitro-EJ* can predict the efficiency of repairing IR-induced DSBs. The repair capacity was determined in NHEJ-deficient cells and their wild type counterparts by scoring the number of DSBs remaining 24 h after X-irradiation using the γH2AX foci assay (Figure 3A). Notably, no difference was reported between all cell lines regarding number of formed γH2AX foci at 1 h post-IR (data not shown). However, results revealed as expected higher numbers of residual γH2AX foci in the NHEJ-deficient cell lines xrs5, XR-1, and MO59J compared to their corresponding wildtype cells (Figure 3B). Importantly, numbers of residual γH2AX foci were found to correlate exclusively with the amount of circular repair products measured by either Southern blotting (Figure 3C, *r*^2^ = 0.906, *P* = 0.013) or transformation assay (Figure 3D, *r*^2^ = 0.958, *P* = 0.004), but not with amount of total end-joining events (Figure S2A, *r*^2^ = 0.649, *P* = 0.100). This indicates that only circular repair product generated in *in vitro-EJ* assay, which represents C-NHEJ efficiency, reflects the *in vivo* DSB repair efficiency after IR.

### The Circular Repair Products Correlate With the Respective Cellular Radiosensitivity

**Figure 4:**
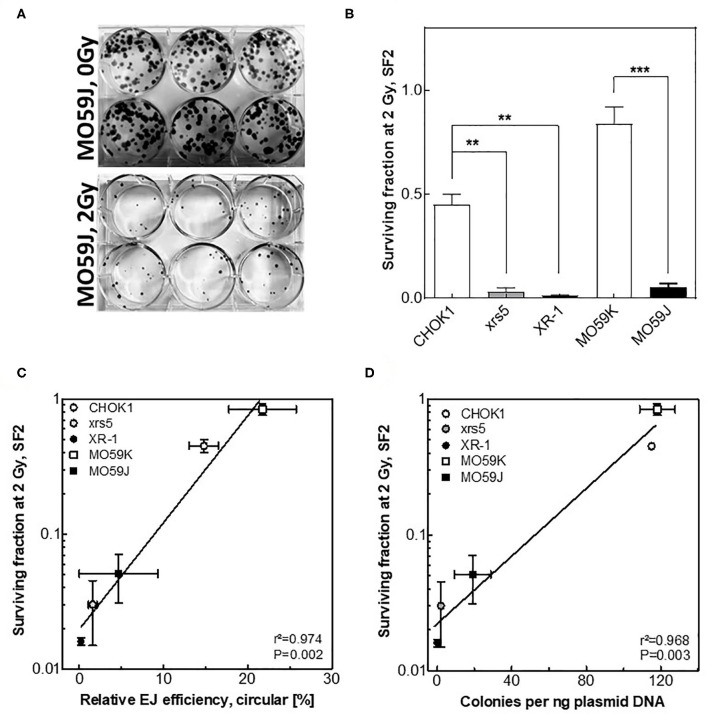
Circular repair products correlate with surviving fraction after IR. **(A)** Depiction of colonies formed in MO59J cells after 0 Gy and 2 Gy treatment on Giemsa-stained 6-well plates. **(B)** Survival fractions measured at 2 Gy using CFA performed in **(A)**. Significance is indicated as **P < 0.001 and ***P < 0.0001. **(C,D)** Negative correlation (Pearson) between the number of residual γH2AX post 2 Gy and relative efficiency of generating circular repair products measured by **(C)** Southern blot or **(D)** transformation assay in the indicated cell lines. Shown are mean ± SEM of at least three independent experiments. Pearson correlation coefficient (*r*^2^) and *p*-value are shown for each comparison.

It has been described that differences observed among individuals in the repair capacities, as indicated by number of residual γH2AX foci, correlate with differences in radiosensitivity (3, 29). Therefore, we tested whether the amount of circular repair products may also reflect indicator for the respective cellular radiosensitivity. To address this issue, cells with different NHEJ efficiencies were irradiated with 2 Gy and survival fractions were measured using colony forming assay (Figure 4A). A huge variation in cell survival was reported between these cell lines after 2 Gy (Figure 4B). Importantly, survival fractions were found to correlate significantly with the amounts of circular products measured using either Southern blotting (Figure 4C, *r*^2^ = 0.974, *P* = 0.002) or transformation assay (Figure 4D, *r*^2^ = 0.968, *P* = 0.003) but again not with the fractions of total end-joining events (Figure S2B, *r*^2^ = 0. 633, *P* = 0.107). These data reveal that the circular repair products measured with the presented *in vitro- EJ* assay can be used as a marker of the cellular radiosensitivity.

### End Joining Is Executed With Huge Efficiency Variations but Primarily *via* C-NHEJ in HNSCC Cell Lines

**Figure 5:**
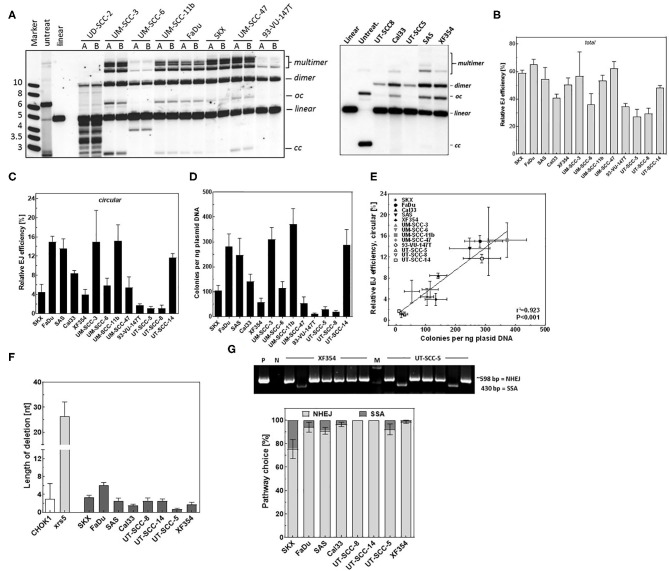
DSB repair is mediated mainly by an accurate NHEJ in HNSCC cells. **(A)** Left and right panels: Southern blot showing the different *in vitro* repair products in the indicated HNSCC cell lines. **(B)** Relative total end joining efficiency measured by Southern blot in the indicated cells. **(C)** Quantification of circular repair products using Southern blot and **(D)** transformation assay in the indicated HNSCC cell lines. **(E)** Correlation between the circular repair products detected using southern and bacterial colonies generated per ng plasmid used for the *in vitro-EJ* assay. Pearson correlation coefficient (*r*^2^) and *p*-value are shown. **(F)** *In vitro* end joining repair junctions were sequenced to determine the deletion length in the indicated cell lines. **(G)** Upper panel: Examples of PCR amplification of repair junctions mediated by XF354 and UT-SCC-5 extracts showing end joining and SSA products. An exact 415-bp fragment indicates a SSA event. P: linearized plasmid. M: DNA marker. Lower panel: Relative fractions of NHEJ events (gray) and SSA (black) in the indicated cell lines. Shown are mean ± SEM of at least three independent experiments.

We sought here to employ the presented *in vitro-EJ* assay to monitor the end-joining efficiencies in tumor cells. Therefore, the *in vitro*-EJ assay was applied in 14 different HNSCC cell lines and end-joining efficiency was determined using both Southern blotting and transformation assay. Notably, a massive DNA degradation (nuclease activity) was seen in the *in vitro-EJ* reaction using CFE of UD-SCC-2 cells (Figure 5A), hampering further analysis of this cell line. The total *in vitro* end joining efficiency obviously varied between the other 13 HNSCC cell lines (Figure 5B). No substantial differences were observed between the cell lines regarding the amount of dimers + multimers (Figure S3A). However, a huge variation was reported in the amount of circular repair products generated in the 13 cell lines using both Southern blot (Figures 5A,C) and transformation assay (Figure 5D). While, very low levels of the circular products were found in 93-VU-147T, UT-SCC-5, and UT-SCC-8, almost 10-fold higher levels were observed in FaDu, SAS, UM-SCC-3, and UM-SCC-11b cells. Again, an excellent correlation between these two methods was reported (Figure 5E, *r*^2^ = 0.923, *P* < 0.001).

Sequencing of a total of 421 repair junctions mediated in HNSCC cells using *in vitro-EJ* reaction revealed in average <4 base pairs deletion, which is similar to the deletion length observed in the wildtype cell line CHO-K1 and far below the level detected for xrs5 cells (Figure 5F). Furthermore, except for SKX cells, SSA was found to be involved in repairing <5% of the induced DSBs (Figure 5G). Overall, these data demonstrate that end-joining in HNSCC cells is not performed by error-prone processes such as Alt-EJ or SSA but almost exclusively by the accurate C- NHEJ, yet with different efficiencies.

### End-Joining Efficiency Partly Reflects the Cellular Radiosensitivity in HNSCC Cell Lines

**Figure 6:**
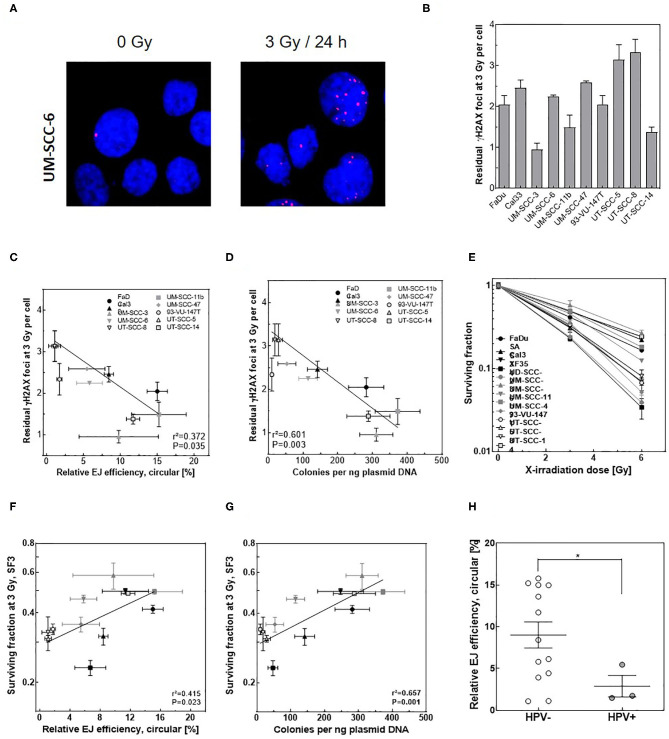
Circular *in vitro* repair product indicates repair capacity and survival after IR in HNSCC cells. **(A)** Representative IF images of γH2AX (red) foci in UM-SSC-6 cells at 24 h pre and post 3 Gy. DAPI was used to counterstain nuclei. **(B)** Quantification of γH2AX foci at 24 h post 3 Gy in the indicated HNSCC cell lines. **(C,D)** Correlation between the number of residual γH2AX foci measured in **(B)** and relative efficiency of generating circular repair products measured by **(C)** Southern blot or **(D)** transformation assay in the indicated cell lines. **(E)** Dose-dependent cell survival in the indicated HNSCC cell lines measured by CFA. **(F,G)** Negative correlation (Pearson) between the survival fractions at 3 Gy (SF3) and relative efficiency of generating circular repair products measured by **(F)** Southern blot or **(G)** transformation assay in the indicated cell lines. Shown are mean ± SEM of at least three independent experiments. Pearson correlation coefficient (*r*^2^) and *p*-value are shown. **(H)** Relative efficiency of C-NHEJ measured by quantifying circular repair products using Southern blot in HPV negative (open circles) and HPV-positive (closed circles) HNSCC cells. Shown are mean ± SEM of at least three independent experiments. Significance is indicated as *p < 0.05.

Finally, we tested whether *in vitro-*EJ assay can predict cellular radiosensitivity in HSNCC cells. To this end, the number of residual γH2AX foci after 24 h post 3 Gy, as an indicator for DSB repair capacity, was scored in the different HNSCC cell lines (Figures 6A,B) and correlated with different *in vitro* end-joining products generated using their cell extracts. No difference between the numbers of formed γH2AX foci in all HNSCC cell lines was reported at 1 h post-IR (data not shown). Instead, the number of residual γH2AX varied between the different HNSCC cells (Figure S3B), indicating different repair capacities. Importantly, the number of residual γH2AX foci correlated significantly with the relative amount of circular repair products measured by transformation assay (Figure 6D, *r*^2^ = 0.53, *P* = 0.007), but neither with the amount of circular products (Figure 6C) nor the amount of dimers + multimers (Figure S3C) as measured by Southern blot.

Confirming our previously published data (37), a huge variation in cellular radiosensitivity was reported for the 13 HNSCC cell lines (Figure 6E). A strong correlation was expectedly seen between the survival fractions at 3 Gy (SF3) and the residual DSBs (Figure S3D, *r*^2^ = 0.911, *P* = 0.001). More importantly, a significant correlation was reported for HNSCC cells between SF3 and the respective individual amount of circular products determined by either Southern blot (Figure 6F, *r*^2^ = 0.415, *P* = 0.023) or transformation assay (Figure 6G, *r*^2^ = 0.657, *P* = 0.001), but not with the amount of dimer + multimer (Figure S3E). However, it is important to note that a substantial scatter was seen for both endpoints. Overall, these data suggest that the described *in vitro-EJ* assay partly predicts both DSB repair efficiency and cellular radiosensitivity in HNSCC cell lines.

## Discussion

In this report, we present a modified *in vitro-EJ* assay which is coupled with a transformation assay to measure DSB repair capacity and radiosensitivity in tumor cells. This system is designed to overcome most of the limitations of the previously reported *in vitro* systems, as it enables the detection of the circular repair products, whose detection was problematic in most of the previously reported systems. Furthermore, the established transformation assay enables specific analysis of the circular repair product, giving a quantitative analysis of this product.

Several *in vitro-EJ* systems have previously been reported using cellular extracts (30, 31, 38–42). However, most of them have many shortcomings. For example, many studies used a nuclear extract as a source of NHEJ proteins, which limits the efficiency of their assays, as NHEJ proteins such as Ku, LigIV are present in cytoplasm and are transported to nuclei in case of DSB signaling (43). One of the major drawbacks of *in vitro-EJ* systems is the requirement of high number of cells for the extract preparation (>1–5 × 10^8^). The present system has provided a microscale assay by scaling down the number of cells required to <10 × 10^6^ cells, with optimum EJ efficiency in different cell lines. This microscale assay can be used for clinical samples and thus the role of DNA DSB repair in tumorigenesis can be studied.

In the current study, we established a powerful in *vitro-EJ* system that facilitates studying not only EJ efficiency but also fidelity as well as pathway switch to the back-up repair pathways such as Alt-EJ and SSA.

Using cell extracts from different NHEJ-deficient mammalian cell lines, we firstly showed that repair products such as dimers and multimers are performed by C-NHEJ, Alt-EJ, or SSA while circular repair products result exclusively from C-NHEJ. This is apparent, because fraction of dimers and multimers was the highest in xrs5 cell line (Figure 1B), whose repair is shifted to Alt-EJ (12, 13, 20) due to the absence of Ku proteins. Interestingly, pushing the end joining reaction toward Alt-EJ by increasing the Mg2^+^ concentration that stimulates the Alt-EJ Ligase LIGIII (Figures 1C,D), rescued the end joining efficiency as evidenced by the increase of dimer and multimer products in DNA-PKcs- or LigIV-deficient cells. In addition to the switch to Alt-EJ, we recapitulated the previously reported significant switch to SSA in xrs5 cells (15). Moreover, the expected repair inaccuracy in NHEJ-deficient cells (12, 13, 20, 36) was verified using our *in vitro-EJ* assay, showing longer deletion length in all NHEJ-deficient cells (Figure 2A). Together these data validate our *in vitro*-EJ assay for detecting total end joining efficiency, fidelity, and repair pathway switch.

Radiosensitivity is governed by the amount of DNA DSBs resulting from exposure and also individual capacity to correctly repair these insults. In this regard, it is important not only to predict the DSB level arising from a given IR dose, but also to consider the DSB repair rate specifically of C-NHEJ, which is the main DSB repair pathway after IR. Given its importance in reflecting the efficiency of C-NHEJ, we developed a transformation assay to specifically detect the circular repair products. We reported an excellent correlation between the amount of circular products determined by Southern blotting and transformation assay (Figure 3D). Importantly, efficiency of generating *in vitro* circular products measured by either Southern blot or transformation assay was found to nicely correlate with (i) the total DSB repair capacity determined via γH2AX foci, and (ii) the cell survival measured by colony forming assay (Figures 4C,D). These data reveal that the presented modified in *vitro-EJ* and transformation assay can be used to detect the *in vivo* repair capacity and cell survival after IR.

For the first time, we showed that HNSCC cell lines may strongly vary in the end-joining efficiency determined by Southern blotting or transformation assay (Figures 5A–D). This variation by a factor of 10 was almost similar to that seen between the wildtype cells CHO K1, MO69K, and their corresponding NHEJ defective cell lines xrs5, XR-1, and MO59J (Figures 1B–D). The reasons for this huge variation in the end-joining efficiency seen for the 13 HNSCC cell lines are not yet clear but may partly arise from differences in the expression of Ku or epidermal growth factor receptor (EGFR), which are both known to affect NHEJ in HNSCC (37, 44).

Having established that the present *in vitro-EJ* assay can detect the switch to Alt-EJ and hence the associated PARP inhibitor (PARPi) radiosensitization, we used it to detect the pathway switch in HNSCC cells. Interestingly, our data demonstrate that the end joining in the HNSCC cells was mediated by a high-fidelity repair mechanism, which avoids the use of Alt-EJ in these cells. This in fact is in line with our previously published data showing that HNSCC cells are not radiosensitized by PARPi (22). However, it is noteworthy that the current findings disagree with the study of Shin et al., who demonstrated an inaccurate end joining repair mechanism in Head and Neck tumor cell lines (17). This contradiction can be explained by the use of different DSB end structures in the two studies. While Shin et al. used compatible cohesive ended DSB, we used in the current study a DSB with a 5′- and a 3′-end, which might need simple filling-in the gap and direct ligation or nucleolytic removal of both overhangs and a simple re-joining process. Alternatively, the different CFE preparations might enable differential nuclease activities.

Importantly, we report a significant correlation between the amount of circular products (measured by either Southern blot or transformation assay) and (i) DSB repair efficiency measured by γH2AX IF assay and (ii) cell survival measured by CFA in HNSCC cells. As expected, this correlation was not observed between the efficiency of dimers/multimers formation and either residual γH2AX or survival fraction, as both dimers and multimers are generated as well by the error-prone mechanisms. Despite the significant correlation between the amount of circular form and residual DSBs or survival fractions after 3 Gy, a substantial scatter was interestingly observed, which can be attributed to the fact that minor C-NHEJ defects could still be compensated by a repair shift to for example the error-free HR mechanism that rescues the survival after IR. This issue needs further investigations.

Another remarkable finding is that three of the HNSCC cell lines used were HPV positive (UD-SCC-2, UM-SCC-47, 93-VU-147T). When compared to HPV negative HNSCC cell lines a clearly lower level of circular repair products was seen for these three cell lines (Figure 6H, *P* = 0.016). These data are in line with the repair deficiency reported in HPV positive cells which render them more radiosensitive compared to HPV negative HNSCC cells (22, 45).

## Concluding Remarks

The presented *in vitro-EJ* assay gives the opportunity not only to estimate the repair capacities of cancer cells in advance of treatment to enable the stratification of patients into radiosensitive or radioresistance categories, but also to identify specific repair deregulation such as a switch to the Alt-EJ or SSA pathway which enables targeting possibilities.

## Data Availability Statement

The original contributions presented in the study are included in the main article/Supplementary Material. Further inquiries can be directed to the corresponding author.

## Author Contributions

SD and KD conducted the experiments and analyzed the results. US led the experiments in Marburg. JD-D, ED, and WM designed the experiments, performed all statistical analyses, and wrote the manuscript. RE-C, CP, and KR reviewed and supervised the manuscript. All authors contributed to the scientific setup of the study, revised the manuscript critically, and they have approved the final version of the manuscript.

## Conflict of Interest

The authors declare that the research was conducted in the absence of any commercial or financial relationships that could be construed as a potential conflict of interest.

## Supplementary Material

Figure S1 **(A)** Depiction of linearized plasmids (pEJ-1200 and pEJSSA-1200) used for the *in vitro-EJ* assay. DSB was induced by restriction enzymes Hind III and PstI which lead to deletion of a 1,200 bp fragment and generation of non-cohesive DSB ends that need processing before being rejoined. **(B)** Upper panel: Schematic representation of the different possible rejoining strategies, including head-to-head (H:H), tail-to-tail (T:T), or head-to-tail (H:T). The indicated primer (P) pairs were employed to differentiate between the different products using PCR. Lower panel: Examples of PCR amplification of repair junctions mediated by cell extracts of the indicated cell lines showing H:H, T:T, and H:T rejoining products. **(C)** Relative band intensity of the corresponding products. **(D)** Southern blot showing the different *in vitro* repair products mediated by incubating linearized pEJ-1200 plasmid with cell extract from FaDu cell line at 25°C (right panel) and 37°C (left panel). **(F)** Southern blot showing the repair products generated with the indicated DNA: protein ratios.

Figure S2 **(A)** Correlation between the number of residual γH2AX post 2 Gy and total relative end joining efficiency measured by quantifying all repair products detected by Southern blot in the indicated cell lines. **(B)** Correlation (Pearson) between total relative *in vitro* end joining efficiency and survival fractions at 2 Gy (SF2) in the indicated cell lines. **(C)** Correlation between the number of residual γH2AX post 2 Gy and SF2 in the indicated cell lines. Shown are mean ± SEM of at least three independent experiments. Pearson correlation coefficient (*r*^2^) and *p*-value are shown for each comparison.

Figure S3 **(A)** Relative efficiency to generate dimer and multimer repair products in the indicated HNSCC cell lines. **(B)** Representative IF images of γH2AX (red) foci at 24 h post 3 Gy in the indicated cell lines. DAPI was used to counterstain nuclei. **(C)** Correlation (Pearson) between relative end joining measured in **(A)** and the number of residual γH2AX post 3 Gy. **(D)** Correlation between the number of residual γH2AX post 3 Gy and SF3 in the indicated HNSCC cell lines. **(E)** Correlation between relative end joining measured in **(A)** and SF3 in the indicated HNSCC cell lines. Shown are mean ± SEM of at least three independent experiments. Pearson correlation coefficient (*r*^2^) and *p*-value are shown for each comparison.
