# Supplementary material for: Establishment of a Transformation Coupled in vitro End Joining Assay to Estimate Radiosensitivity in Tumor Cells
Source: Front Oncol. 2020 Aug 20;10:1480. doi: 10.3389/fonc.2020.01480 (PMC7468517; doi:10.3389/fonc.2020.01480)
Supplement: Figure S1 — (A) Depiction of linearized plasmids (pEJ-1200 and pEJSSA-1200) used for the in vitro-EJ assay. DSB was induced by restriction enzymes Hind III and PstI which lead to deletion of a 1,200 bp fragment and generation of non-cohesive DSB ends that need processing before being rejoined. (B) Upper panel: Schematic representation of the different possible rejoining strategies, including head-to-head (H:H), tail-to-tail (T:T), or head-to-tail (H:T). The indicated primer (P) pairs were employed to differentiate between the different products using PCR. Lower panel: Examples of PCR amplification of repair junctions mediated by cell extracts of the indicated cell lines showing H:H, T:T, and H:T rejoining products. (C) Relative band intensity of the corresponding products. (D) Southern blot showing the different in vitro repair products mediated by incubating linearized pEJ-1200 plasmid with cell extract from FaDu cell line at 25°C (right panel) and 37°C (left panel). (F) Southern blot showing the repair products generated with the indicated DNA: protein ratios. [file Data_Sheet_1.PDF]

Establishment of a transformation coupled *in vitro* end joining assay to measure radiosensitivity in tumor cells

S. Degenhardt [1,§], K. Dreffke [2,§], U. Schötz[2], C. Petersen[3], R. Engenhart-Cabillic[2], K. Rothkamm[1], J. Dahm-Daphi[1,2], E. Dikomey[1,2] and W.Y. Mansour[1, 4*]

[1] Laboratory of Radiobiology & Experimental Radiooncology, University Medical Center Hamburg-Eppendorf, Hamburg, Germany

[2] Department of Radiotherapy and Radiooncology, Philipps-University, Marburg, Germany,

[3] Department of Radiotherapy, University Medical Center Hamburg-Eppendorf, Hamburg, Germany

[4] Mildred Scheel Cancer Career Center, University Medical Center Hamburg-Eppendorf, Hamburg, Germany

[§] These authors contributed equally

* Correspondence:
Dr. Wael Yassin Mansour
Lab. of Radiobiology & Experimental Radiooncology
Campus Science N27
Center of Oncology
University Medical Center Hamburg-Eppendorf
Martinistr. 52
20246 Hamburg, Germany

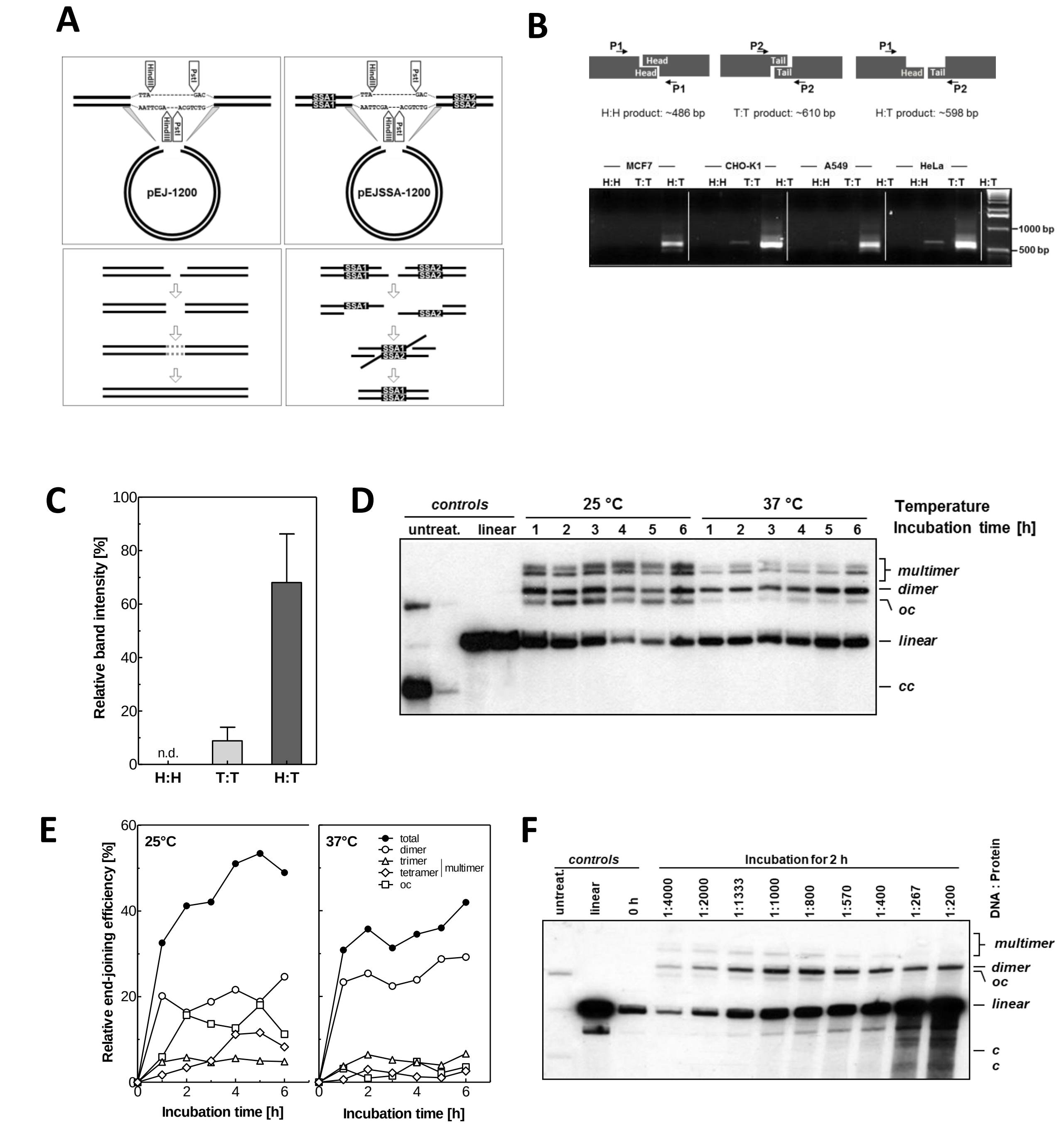

Supplementary Figure S1

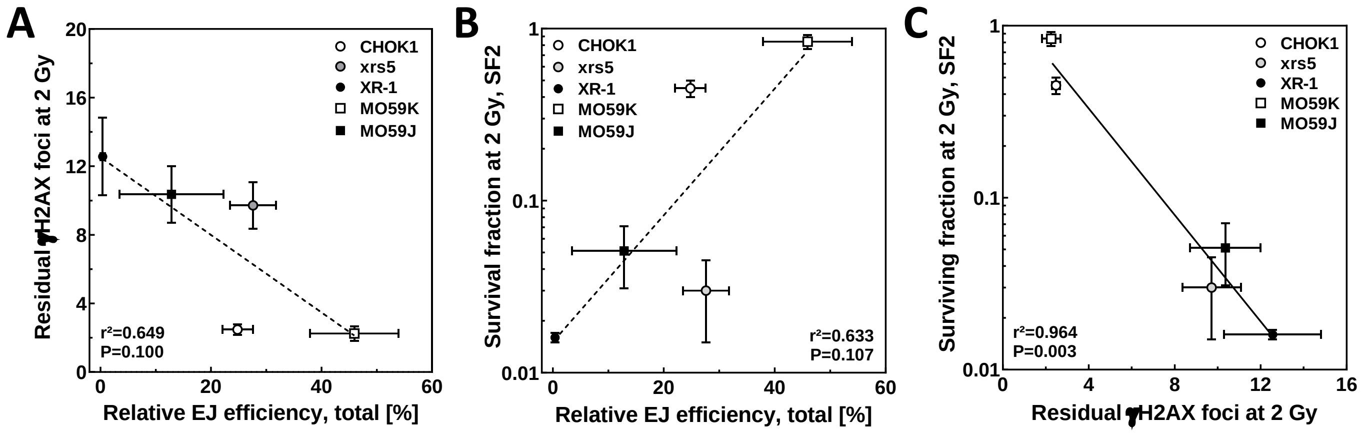

Supplementary Figure S2

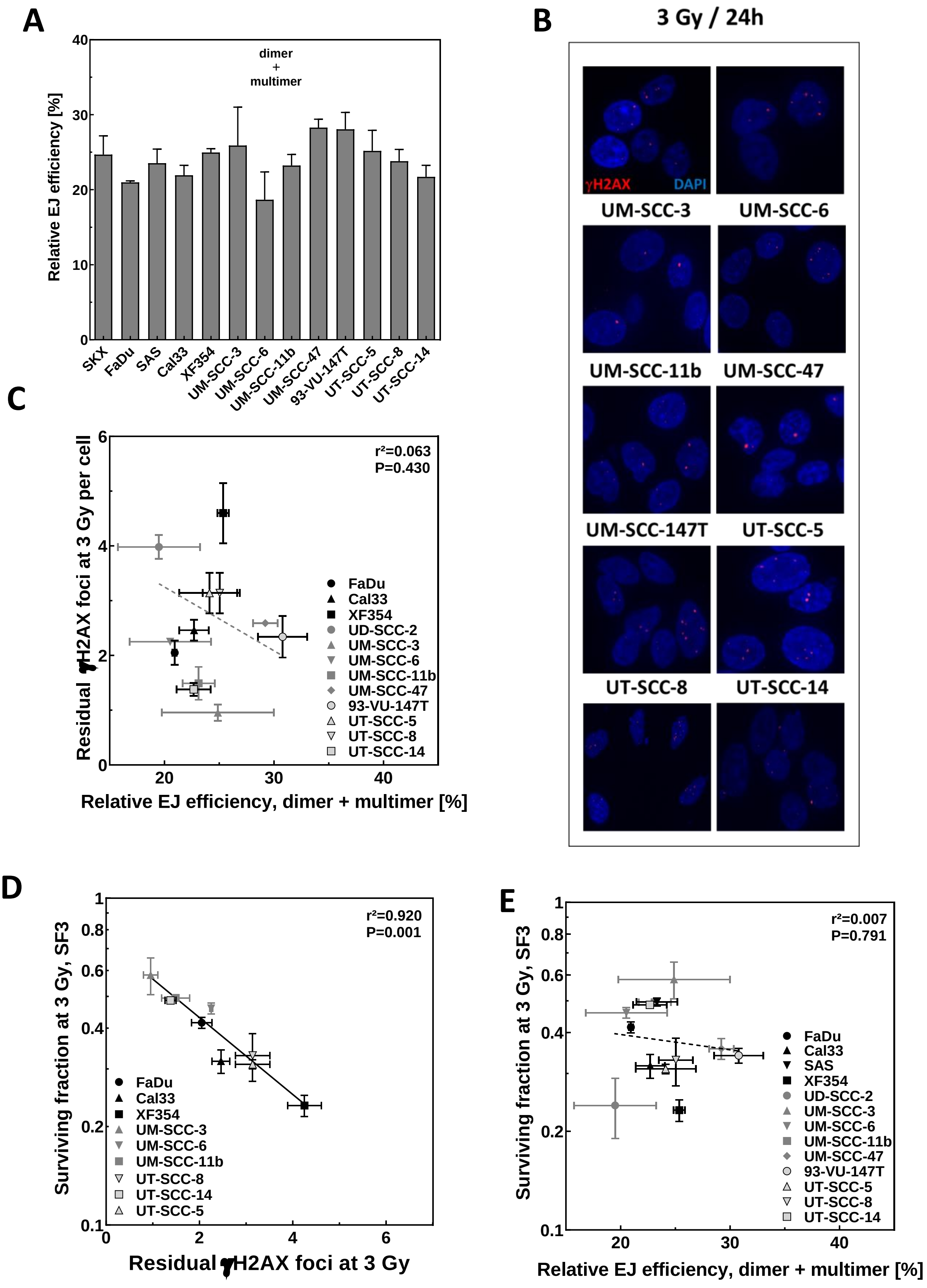

Supplementary Figure S3
